# The impact of the soluble epoxide hydrolase cascade on periodontal tissues

**DOI:** 10.3389/fdmed.2023.1129371

**Published:** 2023-02-01

**Authors:** Henrique Ballassini Abdalla, Thomas E. Van Dyke

**Affiliations:** ^1^ Faculdade São Leopoldo Mandic, Instituto de Pesquisa São Leopoldo Mandic, Campinas, Brazil; ^2^ Clinical and Translational Research, The Forsyth Institute, Cambridge, MA, United States; ^3^ Faculty of Medicine, Harvard University, Boston, MA, United States

**Keywords:** periodontitis, inflammation, lipid mediator, soluble epoxide hydrolase (sEH), soluble epoxide hydrolase (sEH) inhibitors

## Abstract

Periodontitis is a chronic inflammatory disease with complex pathogenesis. Uncontrolled inflammation is driven by the immune system in response to accumulation of oral biofilm that leads to alveolar bone loss, bleeding, increased periodontal probing depth with loss of attachment of the connective tissues to the tooth, and ultimately, tooth loss. Soluble epoxide hydrolase (sEH) is an enzyme that converts epoxy fatty acids (EpFAs) produced by cytochrome P450 (CYP450) to an inactive diol. It has been shown that EpFAs display important features to counteract an exaggerated inflammatory process. Based upon this observation, inhibitors of sEH have been developed and are being proposed as a strategy to regulate proinflammatory lipid mediator production and the chronicity of inflammation. This mini review focuses on the impact of sEH inhibition on periodontal tissues focusing on the mechanisms involved. The interaction between Specialized Pro-Resolving Mediators and sEH inhibition emerges as a significant mechanism of action of sEH inhibitors that was not formerly appreciated and provides new insights into the role SPMs may play in prevention and treatment of periodontitis.

## Introduction

Periodontitis is a chronic inflammatory disease with a complex pathogenesis that encompasses the host immune system and oral microbiome dysbiosis (1–3). The uncontrolled inflammation in the periodontium leads to the destruction of hard and soft tissues and, eventually, tooth loss (4). The unwanted excessive inflammatory reaction in periodontitis is due to the failure of endogenous inflammation resolution pathway activation (5). The cessation of the inflammatory process occurs when a balance between pro-inflammatory and pro-resolution mediators is achieved that determines health or disease (6, 7).

Inflammation is a natural and physiological reaction to injury or infection in all biological systems. This biochemical response is finely orchestrated and well-organized to fight pathogens and to restore homeostasis. It is generally accepted as a vital process for our existence. In an ideal scenario, an inflammatory reaction is self-limiting, characterized by a local increase of protein mediators (cytokines, chemokines) and lipid mediators (LMs) (e.g., prostaglandins and leukotrienes), vascular dilation and enhanced capillary permeability, and leukocyte trafficking and activation (8). The initiation or resolution of inflammation is dictated in large part by the metabolism of polyunsaturated fatty acids (PUFA) by cyclooxygenases (COX), lipoxygenases (LOX), or cytochrome P450 (CYP450) (9, 10).

Eicosanoids, a group of LMs, are oxidized derivates from the metabolism of arachidonic acid (ARA) by oxidative pathways, the COXs, LOXs, or CYP450 enzymes (8, 11). The resulting bioactive molecules, prostanoids, leukotrienes, hydroxyeicosatetraenoic acids (HETEs), epoxyeicosatrienoic acids (EETs), and hydroperoxyeicosatetraenoic acids (HPETEs) are largely generated in inflammation, with distinct biological functions (12). Although much is known about the metabolism of polyunsaturated fatty acids by the cyclooxygenases and lipoxygenases enzymatic pathways and the activities of their downstream metabolites (13), the cytochrome P450 pathways are less understood, and are the center of this mini review. Notably, the EETs, as well as epoxides of other long-chain polyunsaturated fatty acids (EpFA) generated by the cytochrome P450 pathway, are important bioactive lipids with immunomodulatory actions in inflammation (14, 15). Most of these LMs are short-lived due to their rapid metabolization into inactive diols in the presence of soluble epoxide hydrolase (sEH), losing their ability to resolve inflammation (16). Worst, some of their diols contribute to inflammatory cytokine storm and block the initiation of the resolution phase (17). The sEH enzymes are largely found in the liver, brain, spleen, kidney, intestine, and joints (18–20), and high sEH expression was detected in chronic osteolytic inflammatory disorders, such as periodontitis and arthritis (19, 21–23).

Here, this mini review dissects the mechanisms uncovered to date explaining how sEH inhibition impacts the inflammatory process in periodontal tissues, protects against alveolar bone resorption, and speculates possible interactions/synergism between metabolites derived from sEH inhibition and the resolvent lipid mediators (lipoxins, resolvins) in periodontal tissues.

## Periodontitis

Periodontitis is a chronic inflammatory and infectious disease culminating in a dysbiotic dental biofilm that disrupts the homeostasis of the subgingival environment (24). It is the sixth most prevalent disease among inflammatory osteolytic disorders worldwide, representing a significant public health problem (1, 2, 25). Clinically, periodontitis manifests as loss of clinical attachment, alveolar bone resorption, bleeding on probing, and periodontal pockets, and unlike gingivitis, these clinical symptoms are usually permanent. Individual periodontal susceptibility encompasses genetic, behavioral, and environmental factors that regulate the host immune response and generate ideal conditions for pathogenic biofilm microbial colonization (24, 26). Although microbial pathogens are associated with disease progression and severity, the molecular and biological basis of periodontitis is now realized to be the result of an excessive and uncontrolled inflammatory response rather than a classic infection with an exogenous organism(s) (7, 27). This shift in the periodontal disease paradigm began when increased levels of prostaglandin E_2_ (PGE_2_) were found in crevicular fluid of children and adults, and the levels of PGE_2_ were correlated with disease severity. What caught the researcher's attention was that children had higher levels of PGE_2_ than adults, and the capacity of PGE_2_ to provoke periodontal tissue destruction (28–30). In this sense, the inflammation is an essential component of periodontal disease genesis; the tissues are destroyed by the host, not the bacteria.

As a chronic inflammatory disease, periodontitis stimulates a wide range of immune cells, from residents to infiltrating and patrolling cells, that disrupt tissue homeostasis and is characterized by a change in the immune cell composition (31). Additionally, the communication between the osseous and immune systems are intimately interconnected and responsible for bone destruction or remodeling (32–34). Alvarez and colleagues elegantly describe the spatiotemporal profile of the main gingival immune cell composition in ligature induced experimental periodontitis (35). Initially, neutrophils (CD45^+^LY6G^high^LY6C^mid^CD11b^+/−^) are the most abundant leukocyte cells in the gingiva, reaching their peak 24 h after ligature placement, indicating the activation of the innate immune response. This intense infiltration is accompanied by an over-expression of inflammatory cytokines (IL-1β, IL-6, IL-8, IL-12, and TNF-α), giving birth to a hyper-inflammatory phenomenon (36, 37). The transition from innate immunity to adaptive immune response begins on day 3 when tissue-resident macrophages are expanded, and circulating monocytes are recruited to be differentiated into M1-like macrophages (CD45^+^CD64^+^CD11b^+^MHCII^+^) (35). Macrophages are highly plastic cells that can exhibit dual roles in tissue repair or destruction, depending on their microenvironment (36, 38). Particularly, macrophage phenotypes, M1-like (pro-inflammatory subtype) or M2-like subsets (pro-resolving), are temporally associated with the different stages of experimental periodontitis progression (39). Although M1 macrophages are usually associated with an exacerbated inflammatory response, their presence and activation are needed to fight against pathogen invasion during the acute phase. They are implicated in producing several protein and lipid mediators (cytokines, chemokines, lipids mediators), which are fundamental to orchestrating the inflammatory response and guiding the return to tissue homeostasis, in a normal, self-limiting acute inflammatory response (40). On the other hand, resolving macrophages (M2-like) coordinate the resolution process of inflammation by removing dead cells through efferocytosis, producing anti-inflammatory cytokines (e.g., IL-10, IL-4, and TGF-β), counteracting osteoclast activity and boosting osteoblastic functions with augmented cystatin C (39, 40). Moreover, resolving macrophages are well-known synthesizers of Specialized Pro-resolving Mediators (SPMs), a fundamental lipid mediator class switching that defines inflammation termination and resolution stimulation (9, 41).

Failure of the acute response to resolve normally leads to chronicity and chronic inflammatory diseases which include periodontal disease. In experimental periodontitis, T cells (CD45^+^CD3^+^) represent roughly 70% of all cell populations in the gingiva, reaching the peak at day 10 post-ligature (35). Specifically, alveolar bone resorption relies on the imbalance between T-helper type 17 and regulatory T cells (Treg) (40, 42). Although Th17 cells have a physiological immune-protective role in the oral mucosa, their exaggerated activation establishes an interaction with the osteoclast by directly inducing RANKL expression by osteoblasts and periodontal ligament fibroblasts through IL-17A and IL-17F synthesis, ultimately leading to bone loss (34). The CD4^+^ Th17 cells were first described in the early 2000s (43–45). This abnormal reaction is associated with augmented IL-23 levels, from the IL-12 cytokine family (46). Further, transforming growth factor-beta (TGF-beta) primes IL-23R, enhancing the Th17 responsiveness to IL-23 (45), culminating in intense neutrophil transmigration to inflamed sites and RANK/RANKL axis incitement (47). To the contrary, another subset of T cells, Tregs, are regulators of exaggerated inflammatory reactions, maintaining humoral tolerance and reestablishing homeostasis (48). The mainly immunosuppressive Treg features are linked with the release of inhibitory cytokines, such as IL-10, TGF-beta (48), and IL-35 (49), and by dampening dendritic cells *via* the interaction between cytotoxic T-lymphocyte antigen 4 (CTLA4) and cluster of differentiation (CD) 80/86 (48). Curiously, in experimental periodontitis, Tregs from cervical lymph nodes lose their capacity to counteract osteoclastogenic activity, presenting lower expression of Foxp3, and show a Th17-type response (increased IL-17 gene expression) without fully transdifferentiating into Th17-like cells (50).

Endorsing the immunological aspects of periodontal disease progression, inflammatory lipid mediators are dramatically elevated in periodontal tissues and crevicular fluid, such as leukotriene B_4_ (LTB_4_) and prostaglandin E_2_ (PGE_2_) (28–30). Apart from inflammatory lipid mediators, differences in the Specialized Pro-Resolving Mediators (SPMs) and other lipid mediator profiles are associated with the stages of periodontal inflammation (51, 52). Gingival samples from healthy and periodontitis subjects showed distinct lipid profiles in PCA (Principal Component Analysis) of metabolipidomics (51). Notably, none of the SPMs were found to be higher in periodontitis than in healthy subjects, although several pathway markers for omega-6 driven SPMs (e.g., 5-HETE and 15-HETE), D-series resolvins (e.g., 4-HDHA and 7-HDHA), and E-series resolvins [15(S)-HEPE] were higher in periodontitis. Moreover, the resolvin E1 receptor (BLT1) was lower in periodontitis than in healthy subjects’ samples (51). These findings suggest that in periodontitis, there is an effort by the body to re-establish homeostasis and initiate the resolution process through SPM synthesis; however, even though essential pathways seem to be activated, none of the final SPM metabolites were found at physiological levels, enough to exert cell function, and SPM receptor expression was decreased.

## Soluble epoxy hydrolase and its inhibition

John Casida's group led the discovery of soluble epoxide hydrolase in the 1970s, when they described an unknown epoxide hydrolase activity in the soluble fraction of liver homogenates (53–55). Interestingly, the fundamental biological role of sEH is proved by its conservation among species, from chordates to mammalians (56), and it is mostly expressed in the liver, kidney, intestine, brain, and endothelial cells (57).

Soluble epoxide hydrolase was found to be essential for the hydrolysis of the epoxy fatty acids. The epoxy fatty acids are generated by polyunsaturated fatty acid metabolism [including ARA, linoleic acid (LA), eicosapentaenoic acid (EPA), docosahexaenoic acid (DHA), docosapentaenoic acid (DPA)] through the enzymatic activity of cytochrome P450, resulting in lipid mediators with a broad spectrum of biological functions at the systemic and cellular levels (58). The epoxidized metabolites are primarily anti-inflammatory and resolution lipid mediators, such as epoxyeicosatrienoic acids (EETs) from omega-6 ARA, epoxyeicosatrienoic acids (EEQs) from omega-3 EPA, and epoxydocosapentaenoic acids (EDPs) from omega-3 DHA (Wagner et al., 2017). However, in the presence of sEH (their principal regulatory enzyme), these epoxy metabolites are rapidly transformed into inactive diols, which could also possess pro-inflammatory functions (59).

In this regard, targeted inhibition of sEH during the inflammatory process, and consequently, enhancement of epoxy fatty acid bioavailability, offers an attractive strategy for inflammation control. The first inhibitors designed were too unstable for *in vivo* experiments (60). With the advent of crystallographic studies and the discovery of dicyclohexyl urea as a reversible inhibitor of soluble epoxide hydrolase (61), the next generation of inhibitors was produced with higher efficacy, stability, pharmacokinetics, and minor off-target activity (62). Since then, many studies have been carried out in several inflammatory models with promising results. Below, we summarize the findings on soluble epoxide hydrolase inhibition in periodontal tissues.

## Inhibition of soluble epoxy hydrolase in periodontal tissues and *in vitro* assays

The pharmacological inhibition of soluble epoxide hydrolase and its impact on inflammatory, autoimmune, and pain disorders has been widely explored (63–66). Nevertheless, its application in periodontitis or other orofacial conditions is new (18, 21–23, 67). There are only a few studies involving the inhibition of the soluble epoxide hydrolase enzyme in periodontal disease (21–23); therefore, we will address them in detail. Still, in our bibliographic search, we found only one article that shows the impact of EETs on osteoclasts (68) and another on fibroblasts (69), although both are not focused on oral tissues.

**Figure 1:**
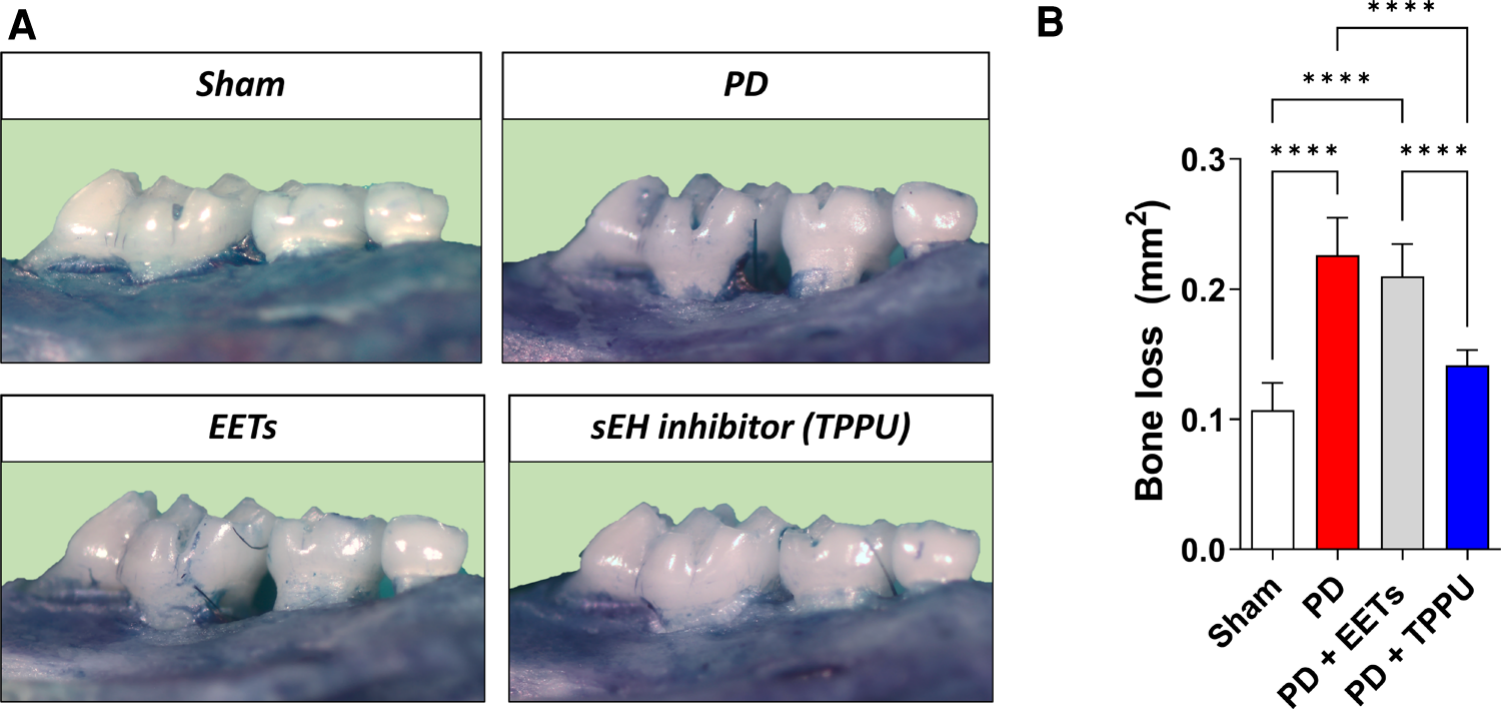
Inhibition of soluble epoxide hydrolase prevents alveolar bone loss in experimental periodontitis in mice. (**A**) Representative images from a palatal view of maxillary molars. TPPU was used as the soluble epoxide hydrolase inhibitor. (**B**) Bone loss was quantified as the area between the cementum-enamel junction and the alveolar bone. PD, periodontal disease; EETs, epoxyeicosatrienoic acids. ****P < 0.0001. The data are expressed as mean ± S.D; *n* = 5 animals per group.

Trindade-da-Silva and colleagues initially demonstrated the protective effect of soluble epoxide hydrolase inhibitors (TPPU) on alveolar bone resorption in experimental periodontitis induced by *Aggregatibacter actinomycetemcomitans* (*Aa*), as exemplified in Figure 1 in a ligature-induced periodontitis model (21). The potential bacteriostatic effect of the sEH inhibitor was discarded when no changes in *Aa*’ growth were found in the presence of TPPU. Subsequently, by measuring the distance between the cemento–enamel junction and the alveolar bone crest, the researchers showed that by inhibiting soluble epoxide hydrolase, lower bone loss in infected animals was detected, altering the phenotype of experimental periodontitis. Interestingly, treatment with EETs, one of the CYP450 metabolite branches that is inactivated by sEH, did not prevent bone loss. Additionally, the treatment with sEH and EETs concomitantly, did not result in a greater prevention of bone loss. Corroborating evidence was provided by genetic inhibition of soluble epoxide hydrolase by gene KO, which showed reduced bone loss, recapitulating the previous observations from the pharmacological inhibition by TPPU (21). Mechanistically, pharmacological inhibition and genetic ablation decreased activation of the RANK/RANKL/OPG axis in gingival tissue. In agreement, the reduced protein expression of MCP-1 (monocyte chemotactic protein 1), a vital monocyte recruiter associated with lower levels of F4/80 (EGF-like module-containing mucin-like hormone receptor-like 1) in the gingiva, endorses that the protective effect of sEH inhibition is related to the regulation of the exaggerated inflammation and the immune system response (21). The decreased inflammatory process was tracked by two essential downstream stress kinases, mitogen-activated protein kinase phosphorylation (p38 and JNK 1/2), which ultimately led to nuclear factor kappa B (NFκB) activation (70). Animals treated with TPPU, TPPU and EETs, or in sEH KOs, showed greatly reduced phosphorylation of p38 and JNK 1/2. Finally, pharmacological sEH inhibition and knockout animals (sEH^−^/^−^) showed inhibition of phosphorylation of the ER stress sensor (PERK, protein kinase RNA-like ER kinase); eIF2α, eukaryotic initiation factor 2α; IRE1, inositol-requiring enzyme 1; sXBP1, spliced X-box binding protein 1 and associated apoptosis (c-Caspase-3 and immunoglobulin binding protein) (21).

Napimoga and collaborators, in a succeeding study by the same research group, showed that inflamed gingival tissue induced by experimental periodontitis expressed higher levels of sEH than control animals. Pharmacological inhibition of sEH dampened this expression, and correlated with lessening disease severity (22). Using an RNA array to explore the innate and adaptive immune systems, sEH inhibition diminished the expression of toll-like receptors 1 and 9 (Trl1 and Trl9), which play a crucial role in inflammatory cytokine release upon triacylated lipopeptide recognition (71) and activation of osteoclastic functions (72). T cells were also affected. The expression of Cd8 and Cd4 was diminished, as well as Cd40l, interferon-alpha2 (Ifnα2), and interferon-beta (Ifnβ) (21). Downregulation of Cd40l impairs B-cell activation and, therefore, the production of IL-2, IL-6, and TNF-alpha (73). The signal transducer and activator of transcription 4 (Stat4) is a factor that contributes to IL-12, IL-23, and IFN-1 production, in addition to differentiating Th1 and Th17 cells (74), which was also reduced by sEH inhibition. These findings reinforce the concept that by inhibiting sEH, the unwanted lymphocyte response is managed, as also demonstrated in a collagen-induced model of arthritis (19), preventing osteoclastogenic activity in the periodontium (21) and knee joint (19).

Recently, Abdalla and coworkers thoroughly characterized the impact of the sEH/EET axis on gingival macrophage plasticity in experimental periodontitis in mice. The work revealed for the first time that pharmacological inhibition of sEH fosters communication between epoxy fatty acid metabolites, increasing the levels of Specialized Pro-Resolving Mediators [e.g., resolvin (Rv) E-series and lipoxins] in saliva, as well as their respective receptors in the gingival tissues (23).

**Figure 2:**
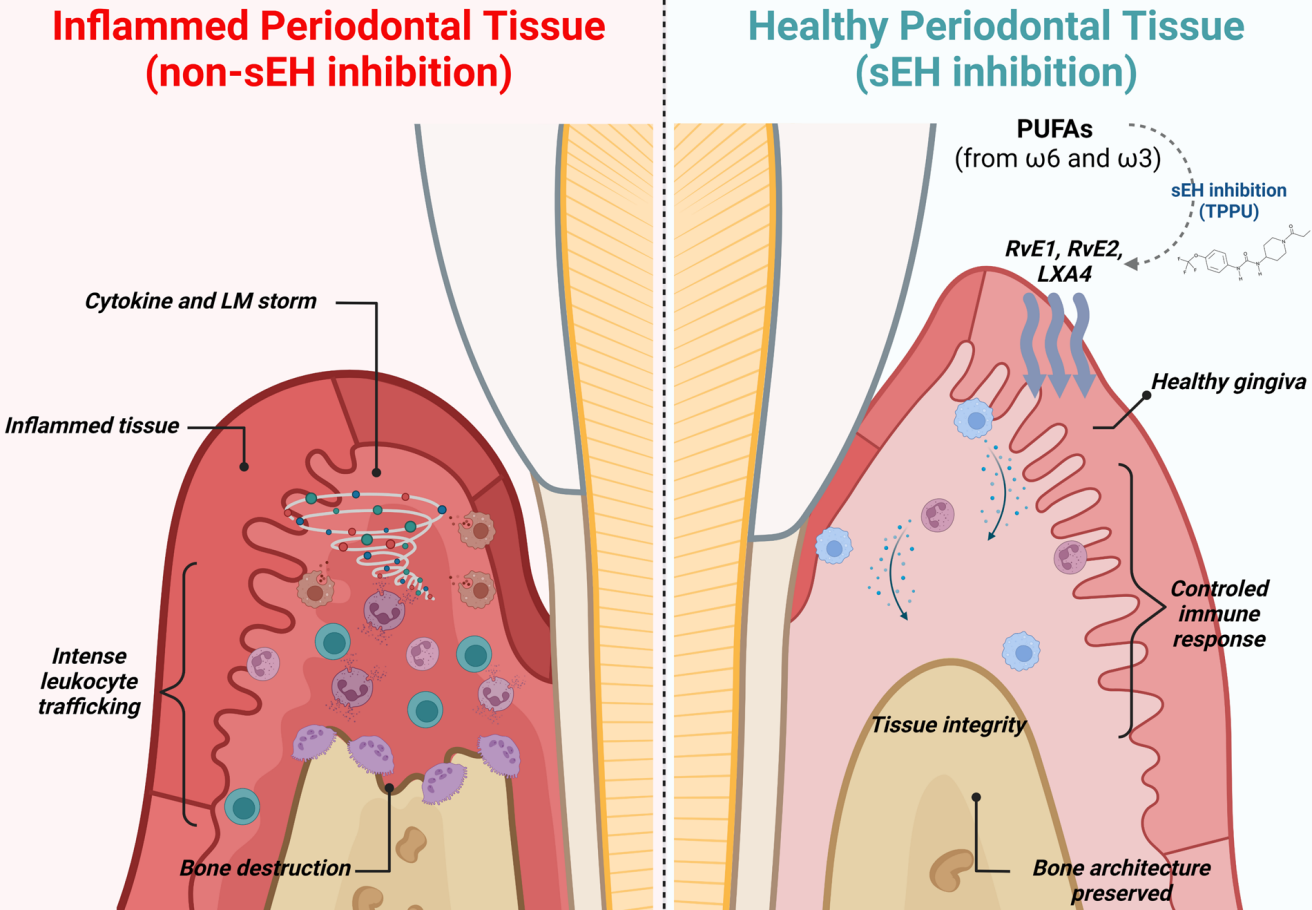
Immune modulation and lipid mediator synthesis induced by sEH inhibition in experimental periodontitis. During periodontitis, the immune system drives an unwanted and uncontrolled inflammatory reaction, leading to an intense release of inflammatory cytokines, chemokines, and lipid mediators, ultimately leading to alveolar bone loss, gingival tissue damage, and increased probing depth (left panel). Pharmacological inhibition of sEH improves the bioavailability of epoxy fatty acids (EpFAs), shifting polyunsaturated fatty acid (PUFA) metabolism and favoring production of Specialized Pro-Resolving Mediators. Further, macrophages undergo phenotypic reprogramming towards resolving and repairing features associated with releasing anti-inflammatory cytokines. Innate immunity is controlled and well-orchestrated. Finally, inhibition of sEH prevents osteoclastic activation, preventing alveolar bone loss.

Mechanistically, pharmacological sEH inhibition suppressed alveolar bone loss *via* actions on inflammatory osteolytic factors, such as Il17a and RANKL. In metabolipidomic analyses, soluble epoxide hydrolase inhibitor treated animals showed lipid profiles that were distinct from experimental periodontitis and control animals in two-dimensional and three-dimensional Principal Component Analyses. The foremost lipid mediators enhanced by sEH inhibition were RvE1, RvE2, and LXA_4_, well-known SPMs with robust immunoresolvent features that guide healing. Moreover, 20-hydroxy LTB_4_ was enhanced, inferring an inactivation of LTB_4_, a critical inflammatory lipid mediator. Further, the Specialized Pro-Resolving Mediator receptors (LTB4R1, CMKLR1/ChemR23, and ALX/FPR2) were also found to increase in gingival tissue, suggesting greater effectiveness of SPM activity at the site of inflammation. In macrophages, the pharmacological inhibition of soluble epoxide hydrolase stimulated a dynamic transcriptional reprogramming of inflammatory macrophages toward resolving macrophages (characterized by CD11c^+^/CD206^+^ double-positive cells in the CD45^+^/CD11b^+^/CD64^+^ macrophage population), associated with reduced expression of Il1β, TNFα, Il12, and Nos2. Finally, *in vitro* assays revealed that sEH inhibition and EET treatment triggered SPM release in bone marrow derived macrophages (BMDMs) in both inflammatory and resolvent macrophages (23). These findings are summarized in Figure 2.

The direct influence of the EETs/sEH/DHET axis on osteoclast differentiation and activity was explored *in vitro* using BMMCs (Bone marrow mononuclear cells) and RAW264.7 murine cells (68). Authors showed that DHETs, the inactive diol form of EETs, could not reduce TRAP-positive cells, but increased their number. Differently, treatment with EETs or sEH inhibitors (TPPU) significantly diminished the number of multinucleated TRAP-positive cells. Likewise, bone resorption pits were hardly impaired by EETs and sEH inhibition, as well as expression of RANK, TRAP, cathepsin K (CK), and matrix metalloproteinase (MMP)-9. Further, in an osteoblast precursor cell line (MC3T3), EETs reduced the ratio between RANKL:OPG (68). In TGF-β1-induced activation of murine fibroblasts (NIH3T3), EETs attenuate cell activation by impairing the expression of collagen, smooth muscle alpha-actin (α-SMA), and proliferating cell nuclear antigen (PCNA) in a peroxisome proliferation activated receptor γ (PPARγ) dependent-manner (69).

## Conclusions and perspective

The pharmacological inhibition of sEH has shown impressive results in inflammatory diseases and has been the subject of extensive research. Concerning the dental medicine area, including painful orofacial conditions and periodontal disease, a few studies have been conducted, revealing promising findings. As a note, sEH inhibitors are in the clinical development phase, making them a promising forthcoming therapeutic strategy. Nevertheless, a profound molecular mechanistic analysis of how sEH inhibition acts through the immune system must be carried out. Future research should deeply analyze the impact of sEH inhibition on immune system cells and how they respond in its absence. Nevertheless, recent findings demonstrate that the inhibition of sEH influences the production of SPMs (omega-3 and -6 fatty acids metabolites from CYP450), which paves the way for a new perspective on its mechanism of action, as well as pharmacological implications, as they boost resolution pathways of inflammation rather than silencing them.

## References

[1] Hajishengallis G Chavakis T. Local and systemic mechanisms linking periodontal disease and inflammatory comorbidities. Nat Rev Immunol. (2021) 21(7):426–40. 10.1038/s41577-020-00488-6 33510490 PMC7841384

[2] Lamont RJ Hajishengallis G. Polymicrobial synergy and dysbiosis in inflammatory disease. Trends Mol Med. (2015) 21(3):172–83. 10.1016/j.molmed.2014.11.004 25498392 PMC4352384

[3] Alvarez C Monasterio G Cavalla F Córdova LA Hernández M Heymann D Osteoimmunology of oral and maxillofacial diseases: translational applications based on biological mechanisms. Front Immunol. (2019) 10:1664. 10.3389/fimmu.2019.01664 31379856 PMC6657671

[4] Van Dyke TE Sima C. Understanding resolution of inflammation in periodontal diseases: is chronic inflammatory periodontitis a failure to resolve? Periodontol 2000. (2020) 82(1):205–13. 10.1111/prd.12317 31850636

[5] Van Dyke TE. Proresolving lipid mediators: potential for prevention and treatment of periodontitis. J Clin Periodontol. (2011) 38(Suppl 11):119–25. 10.1111/j.1600-051X.2010.01662.x 21323709

[6] Balta MG Papathanasiou E Blix IJ Van Dyke TE. Host modulation and treatment of periodontal disease. J Dent Res. (2021) 100(8):798–809. 10.1177/0022034521995157 33655803 PMC8261853

[7] Van Dyke TE. Shifting the paradigm from inhibitors of inflammation to resolvers of inflammation in periodontitis. J Periodontol. (2020) 91(Suppl 1):S19–25. 10.1002/JPER.20-0088 32441774 PMC8142079

[8] Serhan CN. Pro-resolving lipid mediators are leads for resolution physiology. Nature. (2014) 510(7503):92–101. 10.1038/nature13479 24899309 PMC4263681

[9] Panigrahy D Gilligan MM Serhan CN Kashfi K. Resolution of inflammation: an organizing principle in biology and medicine. Pharmacol Ther. (2021) 227:107879. 10.1016/j.pharmthera.2021.107879 33915177

[10] Maddipati KR. Non-inflammatory physiology of “inflammatory” mediators - unalamation, a new paradigm. Front Immunol. (2020) 11:580117. 10.3389/fimmu.2020.580117 33117385 PMC7575772

[11] Wang D Dubois RN. Eicosanoids and cancer. Nat Rev Cancer. (2010) 10(3):181–93. 10.1038/nrc2809 20168319 PMC2898136

[12] Haeggström JZ Funk CD. Lipoxygenase and leukotriene pathways: biochemistry, biology, and roles in disease. Chem Rev. (2011) 111(10):5866–98. 10.1021/cr200246d 21936577

[13] Hoxha M Zappacosta B. CYP-derived eicosanoids: implications for rheumatoid arthritis. Prostaglandins Other Lipid Mediat. (2020) 146:106405. 10.1016/j.prostaglandins.2019.106405 31838196

[14] Zeldin DC. Epoxygenase pathways of arachidonic acid metabolism. J Biol Chem. (2001) 276(39):36059–62. 10.1074/jbc.R100030200 11451964

[15] Hammock BD Wang W Gilligan MM Panigrahy D. Eicosanoids: the overlooked storm in coronavirus disease 2019 (COVID-19)? Am J Pathol. (2020) 190(9):1782–8. 10.1016/j.ajpath.2020.06.010 32650004 PMC7340586

[16] Wang B Wu L Chen J Dong L Chen C Wen Z Metabolism pathways of arachidonic acids: mechanisms and potential therapeutic targets. Signal Transduct Target Ther. (2021) 6(1):94. 10.1038/s41392-020-00443-w 33637672 PMC7910446

[17] Kodani SD Hammock BD. The 2014 bernard B. Brodie award lecture-epoxide hydrolases: drug metabolism to therapeutics for chronic pain. Drug Metab Dispos. (2015) 43(5):788–802. 10.1124/dmd.115.063339 25762541 PMC4407705

[18] Abdalla HB Napimoga MH Teixeira JM Trindade-da-Silva CA Pieroni VL Dos Santos Araújo FSM Soluble epoxide hydrolase inhibition avoid formalin-induced inflammatory hyperalgesia in the temporomandibular joint. Inflammopharmacology. (2022) 30(3):981–90. 10.1007/s10787-022-00965-5 35303234 PMC9578439

[19] Trindade-da-Silva CA Clemente-Napimoga JT Abdalla HB Rosa SM Ueira-Vieira C Morisseau C Soluble epoxide hydrolase inhibitor, TPPU, increases regulatory T cells pathway in an arthritis model. FASEB J. (2020) 34(7):9074–86. 10.1096/fj.202000415R 32400048 PMC7383812

[20] Norwood S Liao J Hammock BD Yang GY. Epoxyeicosatrienoic acids and soluble epoxide hydrolase: potential therapeutic targets for inflammation and its induced carcinogenesis. Am J Transl Res. (2010) 2(4):447–57. PMID: ; PMCID: 20733953 PMC2923867

[21] Trindade-da-Silva CA Bettaieb A Napimoga MH Lee KSS Inceoglu B Ueira-Vieira C Soluble epoxide hydrolase pharmacological inhibition decreases alveolar bone loss by modulating host inflammatory response, RANK-related signaling, endoplasmic Reticulum stress, and apoptosis. J Pharmacol Exp Ther. (2017) 361(3):408–16. 10.1124/jpet.116.238113 28356494 PMC5443319

[22] Napimoga MH Rocha EP Trindade-da-Silva CA Demasi APD Martinez EF Macedo CG Soluble epoxide hydrolase inhibitor promotes immunomodulation to inhibit bone resorption. J Periodontal Res. (2018) 53(5):743–9. 10.1111/jre.12559 29851077 PMC6168335

[23] Abdalla HB Alvarez C Wu YC Rojas P Hammock BD Maddipati KR Soluble epoxide hydrolase inhibition enhances specialized pro-resolving lipid mediator production and promotes macrophage plasticity. Br J Pharmacol. (2022). 10.1111/bph.16009. [Epub ahead of print] PMC10175184 36508312

[24] Papapanou PN Sanz M Buduneli N Dietrich T Feres M Fine DH Periodontitis: consensus report of workgroup 2 of the 2017 world workshop on the classification of periodontal and peri-implant diseases and conditions. J Periodontol. (2018) 89(Suppl 1):S173–82. 10.1002/JPER.17-0721 29926951

[25] Kassebaum NJ Smith AGC Bernabé E Fleming TD Reynolds AE Vos T Global, regional, and national prevalence, incidence, and disability-adjusted life years for oral conditions for 195 countries, 1990-2015: a systematic analysis for the global burden of diseases, injuries, and risk factors. J Dent Res. (2017) 96(4):380–7. 10.1177/0022034517693566 28792274 PMC5912207

[26] Kurgan S Kantarci A. Molecular basis for immunohistochemical and inflammatory changes during progression of gingivitis to periodontitis. Periodontol 2000. (2018) 76(1):51–67. 10.1111/prd.12146 29194785

[27] Van Dyke TE Bartold PM Reynolds EC. The nexus between periodontal inflammation and dysbiosis. Front Immunol. (2020) 11:511. 10.3389/fimmu.2020.00511 32296429 PMC7136396

[28] Offenbacher S Odle BM Gray RC Van Dyke TE. Crevicular fluid prostaglandin E levels as a measure of the periodontal disease status of adult and juvenile periodontitis patients. J Periodontal Res. (1984) 19(1):1–13. 10.1111/j.1600-0765.1984.tb01190.x 6232362

[29] Offenbacher S Odle BM Van Dyke TE. The use of crevicular fluid prostaglandin E2 levels as a predictor of periodontal attachment loss. J Periodontal Res. (1986) 21(2):101–12. 10.1111/j.1600-0765.1986.tb01443.x 2937899

[30] Van Dyke TE Lester MA Shapira L. The role of the host response in periodontal disease progression: implications for future treatment strategies. J Periodontol. (1993) 64(Suppl 8S):792–806. 10.1902/jop.1993.64.8s.792 29539752

[31] Hajishengallis G. Periodontitis: from microbial immune subversion to systemic inflammation. Nat Rev Immunol. (2015) 15(1):30–44. 10.1038/nri3785 25534621 PMC4276050

[32] Arron JR Choi Y. Bone versus immune system. Nature. (2000) 408(6812):535–6. 10.1038/35046196 11117729

[33] Takayanagi H Ogasawara K Hida S Chiba T Murata S Sato K T-cell-mediated regulation of osteoclastogenesis by signalling cross-talk between RANKL and IFN-gamma. Nature. (2000) 408(6812):600–5. 10.1038/35046102 11117749

[34] Ikeuchi T Moutsopoulos NM. Osteoimmunology in periodontitis; a paradigm for Th17/IL-17 inflammatory bone loss. Bone. (2022) 163:116500. 10.1016/j.bone.2022.116500 35870792 PMC10448972

[35] Alvarez C Abdalla H Sulliman S Rojas P Wu YC Almarhoumi R Rve1 impacts the gingival inflammatory infiltrate by inhibiting the T cell response in experimental periodontitis. Front Immunol. (2021) 12:664756. 10.3389/fimmu.2021.664756 34012448 PMC8126725

[36] Ling MR Chapple IL Matthews JB. Peripheral blood neutrophil cytokine hyper-reactivity in chronic periodontitis. Innate Immun. (2015) 21(7):714–25. 10.1177/1753425915589387 26055820

[37] Dutzan N Konkel JE Greenwell-Wild T Moutsopoulos NM. Characterization of the human immune cell network at the gingival barrier. Mucosal Immunol. (2016) 9(5):1163–72. 10.1038/mi.2015.136 26732676 PMC4820049

[38] Chazaud B. Macrophages: supportive cells for tissue repair and regeneration. Immunobiology. (2014) 219(3):172–8. 10.1016/j.imbio.2013.09.001 24080029

[39] Viniegra A Goldberg H Çil Ç Fine N Sheikh Z Galli M Resolving macrophages counter osteolysis by anabolic actions on bone cells. J Dent Res. (2018) 97(10):1160–9. 10.1177/0022034518777973 29993312 PMC6169030

[40] Garlet GP Giannobile WV. Macrophages: the bridge between inflammation resolution and tissue repair? J Dent Res. (2018) 97(10):1079–81. 10.1177/0022034518785857 29993304 PMC6169033

[41] Dalli J Serhan C. Macrophage proresolving mediators-the when and where. Microbiol Spectr. (2016) 4(3):10.1128/microbiolspec.MCHD-0001-2014. 10.1128/microbiolspec.MCHD-0001-2014 27337457 PMC4922532

[42] Dutzan N Kajikawa T Abusleme L Greenwell-Wild T Zuazo CE Ikeuchi T A dysbiotic microbiome triggers TH17 cells to mediate oral mucosal immunopathology in mice and humans. Sci Transl Med. (2018) 10(463):eaat0797. 10.1126/scitranslmed.aat0797 30333238 PMC6330016

[43] Ivanov II McKenzie BS Zhou L Tadokoro CE Lepelley A Lafaille JJ The orphan nuclear receptor RORgammat directs the differentiation program of proinflammatory IL-17+ T helper cells. Cell. (2006) 126(6):1121–33. 10.1016/j.cell.2006.07.035 16990136

[44] Veldhoen M Hocking RJ Atkins CJ Locksley RM Stockinger B. TGFbeta in the context of an inflammatory cytokine milieu supports de novo differentiation of IL-17-producing T cells. Immunity. (2006) 24(2):179–89. 10.1016/j.immuni.2006.01.001 16473830

[45] Mangan PR Harrington LE O'Quinn DB Helms WS Bullard DC Elson CO Transforming growth factor-beta induces development of the T(H)17 lineage. Nature. (2006) 441(7090):231–4. 10.1038/nature04754 16648837

[46] Oppmann B Lesley R Blom B Timans JC Xu Y Hunte B Novel p19 protein engages IL-12p40 to form a cytokine, IL-23, with biological activities similar as well as distinct from IL-12. Immunity. (2000) 13(5):715–25. 10.1016/S1074-7613(00)00070-4 11114383

[47] Pan W Wang Q Chen Q. The cytokine network involved in the host immune response to periodontitis. Int J Oral Sci. (2019) 11(3):30. 10.1038/s41368-019-0064-z 31685798 PMC6828663

[48] Vignali DA Collison LW Workman CJ. How regulatory T cells work. Nat Rev Immunol. (2008) 8(7):523–32. 10.1038/nri2343 18566595 PMC2665249

[49] Collison LW Workman CJ Kuo TT Boyd K Wang Y Vignali KM The inhibitory cytokine IL-35 contributes to regulatory T-cell function. Nature. (2007) 450(7169):566–9. 10.1038/nature06306 18033300

[50] Alvarez C Suliman S Almarhoumi R Vega ME Rojas C Monasterio G Regulatory T cell phenotype and anti-osteoclastogenic function in experimental periodontitis. Sci Rep. (2020) 10(1):19018. 10.1038/s41598-020-76038-w 33149125 PMC7642388

[51] Ferguson B Bokka NR Maddipati KR Ayilavarapu S Weltman R Zhu L Distinct profiles of specialized pro-resolving lipid mediators and corresponding receptor gene expression in periodontal inflammation. Front Immunol. (2020) 11:1307. 10.3389/fimmu.2020.01307 32670289 PMC7330171

[52] Elabdeen HR Mustafa M Szklenar M Rühl R Ali R Bolstad AI. Ratio of pro-resolving and pro-inflammatory lipid mediator precursors as potential markers for aggressive periodontitis. PLoS One. (2013) 8(8):e70838. 10.1371/journal.pone.0070838 23951021 PMC3741366

[53] Gill SS Hammock BD Yamamoto I Casida JE. Preliminary chromatographic studies on the metabolites and photodecomposition products of the juvenoid 1-(4'-ethylphenoxy)-6,7-epoxy-3,7-dimethyl-2-octene. In: Menn JJ Beroza M, editors. Insect juvenile hormones: Chemistry and action. New York: Academic Press (1972). p. 177–89.

[54] Gill SS Hammock BD Casida JE. Mammalian metabolism and environmental degradation of the juvenoid 1-(4'-ethylphenoxy)-3,7-dimethyl-6,7-epoxy-trans-2-octene and related compounds. J Agric Food Chem. (1974) 22(3):386–95. 10.1021/jf60193a058 4840500

[55] Hammock BD Gill SS Stamoudis V Gilbert LI. Soluble mammalian epoxide hydratase: action on juvenile hormone and other terpenoid epoxides. Comp Biochem Physiol B. (1976) 53(2):263–5. 10.1016/0305-0491(76)90045-6 1253563

[56] Harris TR Hammock BD. Soluble epoxide hydrolase: gene structure, expression and deletion. Gene. (2013) 526(2):61–74. 10.1016/j.gene.2013.05.008 23701967 PMC3733540

[57] Enayetallah AE French RA Thibodeau MS Grant DF. Distribution of soluble epoxide hydrolase and of cytochrome P450 2C8, 2C9, and 2J2 in human tissues. J Histochem Cytochem. (2004) 52(4):447–54. 10.1177/002215540405200403 15033996

[58] Dyall SC Balas L Bazan NG Brenna JT Chiang N da Costa Souza F Polyunsaturated fatty acids and fatty acid-derived lipid mediators: recent advances in the understanding of their biosynthesis, structures, and functions. Prog Lipid Res. (2022) 86:101165. 10.1016/j.plipres.2022.101165 35508275 PMC9346631

[59] Spector AA. Arachidonic acid cytochrome P450 epoxygenase pathway. J Lipid Res. (2009) 50:S52–6. 10.1194/jlr.R800038-JLR200 18952572 PMC2674692

[60] Mullin CA Hammock BD. Chalcone oxides–potent selective inhibitors of cytosolic epoxide hydrolase. Arch Biochem Biophys. (1982) 216(2):423–39. 10.1016/0003-9861(82)90231-4 7114846

[61] Morisseau C Goodrow MH Dowdy D Zheng J Greene JF Sanborn JR Potent urea and carbamate inhibitors of soluble epoxide hydrolases. Proc Natl Acad Sci U S A. (1999) 96(16):8849–54. 10.1073/pnas.96.16.8849 10430859 PMC17696

[62] Morisseau C Hammock BD. Impact of soluble epoxide hydrolase and epoxyeicosanoids on human health. Annu Rev Pharmacol Toxicol. (2013) 53:37–58. 10.1146/annurev-pharmtox-011112-140244 23020295 PMC3578707

[63] Wagner KM McReynolds CB Schmidt WK Hammock BD. Soluble epoxide hydrolase as a therapeutic target for pain, inflammatory and neurodegenerative diseases. Pharmacol Ther. (2017) 180:62–76. 10.1016/j.pharmthera.2017.06.006 28642117 PMC5677555

[64] Fishbein A Hammock BD Serhan CN Panigrahy D. Carcinogenesis: failure of resolution of inflammation? Pharmacol Ther. (2021) 218:107670. 10.1016/j.pharmthera.2020.107670 32891711 PMC7470770

[65] Wang Y Wagner KM Morisseau C Hammock BD. Inhibition of the soluble epoxide hydrolase as an analgesic strategy: a review of preclinical evidence. J Pain Res. (2021) 14:61–72. 10.2147/JPR.S241893 33488116 PMC7814236

[66] Shan J Hashimoto K. Soluble epoxide hydrolase as a therapeutic target for neuropsychiatric disorders. Int J Mol Sci. (2022) 23(9):4951. 10.3390/ijms23094951 35563342 PMC9099663

[67] Teixeira JM Abdalla HB Basting RT Hammock BD Napimoga MH Clemente-Napimoga JT. Peripheral soluble epoxide hydrolase inhibition reduces hypernociception and inflammation in albumin-induced arthritis in temporomandibular joint of rats. Int Immunopharmacol. (2020) 87:106841. 10.1016/j.intimp.2020.106841 32736189 PMC8015648

[68] Guan H Zhao L Cao H Chen A Xiao J. Epoxyeicosanoids suppress osteoclastogenesis and prevent ovariectomy-induced bone loss. FASEB J. (2015) 29(3):1092–101. 10.1096/fj.14-262055 25466887

[69] Tao JH Liu T Zhang CY Zu C Yang HH Liu YB Epoxyeicosatrienoic acids inhibit the activation of murine fibroblasts by blocking the TGF-β1-Smad2/3 signaling in a PPARγ-dependent manner. Oxid Med Cell Longev. (2022) 2022:7265486. 10.1155/2022/7265486 36275905 PMC9584742

[70] Nandipati KC Subramanian S Agrawal DK. Protein kinases: mechanisms and downstream targets in inflammation-mediated obesity and insulin resistance. Mol Cell Biochem. (2017) 426(1-2):27–45. 10.1007/s11010-016-2878-8 27868170 PMC5291752

[71] Jin MS Kim SE Heo JY Lee ME Kim HM Paik SG Crystal structure of the TLR1-TLR2 heterodimer induced by binding of a tri-acylated lipopeptide. Cell. (2007) 130(6):1071–82. 10.1016/j.cell.2007.09.008 17889651

[72] Matsumoto C Oda T Yokoyama S Tominari T Hirata M Miyaura C Toll-like receptor 2 heterodimers, TLR2/6 and TLR2/1 induce prostaglandin E production by osteoblasts, osteoclast formation and inflammatory periodontitis. Biochem Biophys Res Commun. (2012) 428(1):110–5. 10.1016/j.bbrc.2012.10.016 23063683

[73] Figueredo CM Lira-Junior R Love RM. T and B cells in periodontal disease: new functions in A complex scenario. Int J Mol Sci. (2019) 20(16):3949. 10.3390/ijms20163949 31416146 PMC6720661

[74] Cope AP Schulze-Koops H Aringer M. The central role of T cells in rheumatoid arthritis. Clin Exp Rheumatol. (2007) 25(5 Suppl 46):S4–11. PMID: 17977483

